# Evaluation of the Tobacco-Use Prevention Education (TUPE) program in California

**DOI:** 10.1371/journal.pone.0206921

**Published:** 2018-11-02

**Authors:** Sara B. McMenamin, Sharon E. Cummins, Yue-Lin Zhuang, Anthony C. Gamst, Carlos G. Ruiz, Antonio Mayoral, Shu-Hong Zhu

**Affiliations:** 1 Department of Family Medicine and Public Health, University of California San Diego, La Jolla, California, United States of America; 2 Moores Cancer Center, University of California San Diego, La Jolla, California, United States of America; 3 Department of Mathematics, University of California San Diego, La Jolla, California, United States of America; Leibniz Institute for Prevention Research and Epidemiology BIPS, GERMANY

## Abstract

**Background and aims:**

The California Tobacco-Use Prevention Education (TUPE) program promotes the use of evidence-based tobacco-specific prevention and cessation programs for adolescents within the school setting. Through a competitive grant process, schools are funded to provide programs for grades 6–12. This research evaluates the association between TUPE funding and tobacco prevention activities and tobacco use prevalence.

**Methods:**

This study utilized two data sources: (1) 2016 California Educator Tobacco Survey (CETS), and (2) 2015–2016 California Student Tobacco Survey (CSTS). The CETS collected data from educators about school prevention efforts, priority of tobacco prevention, and confidence in addressing tobacco issues with students. A total of 3,564 educators from 590 schools participated in CETS. The CSTS collected data from 8^th^, 10^th^, and 12^th^ graders in California on their exposure to, attitudes about, and utilization of tobacco products. A total of 47,981 students from 117 schools participated in CSTS.

**Results:**

This study found that TUPE-funded schools were more likely to provide tobacco-specific health education programs, to place a priority on tobacco-prevention efforts, and to prepare educators to address tobacco use than non-TUPE schools. Educators at both types of schools felt better prepared to talk with students about traditional tobacco products than about emerging products such as e-cigarettes. Overall, students at TUPE-funded schools were more likely to report receiving anti-tobacco messages from school-based programs than those at non-TUPE schools. The former were also less likely to use tobacco products, even when the analysis controlled for demographics and school-level characteristics (OR = 0.82 [95% CI = 0.70–0.96]).

**Conclusions:**

TUPE funding was associated with an increase in schools’ tobacco-specific prevention activities and these enhanced activities were associated with lower tobacco use among students. This study also found that education and prevention efforts regarding emerging tobacco products need to be strengthened across all schools.

## Introduction

Adolescence is a period of “heightened vulnerability” to initiating tobacco use.[1,2] The Tobacco-Use Prevention Education (TUPE) program, administered by the California Department of Education, addresses this challenge by promoting evidence-based tobacco-specific education and cessation programs for adolescents in a school setting. Established in 1989, California’s TUPE program started as an entitlement to public kindergarten through 12^th^ grade (K-12) schools. It currently funds programs for grades 6–12 through competitive grants.[3,4]

A fully implemented TUPE program consists of three components: (1) establishment and enforcement of tobacco-free campus policies, (2) participation in the California Healthy Kids Survey (CHKS), and (3) provision of evidence-based educational curricula for tobacco-use prevention and cessation. There are two mechanisms for TUPE funding. School districts that implement components (1) and (2) receive a flat fee of $1,500 per year (i.e. Tier 1). School districts, direct-funded charter schools, county offices of education, or consortia may apply for Tier 2 funding, which is more substantial ($18 per pupil per year, based on the average daily attendance) and goes directly to schools to implement all three components.[5] Because school systems differ in size, and proposals differ in scope, Tier 2 TUPE grants can vary from $30,000 to nearly $2 million for a three-year cycle (average = $725,000).[5]

Previous research has examined the effect of school-based tobacco-use prevention programs. Some research concluded there is no consistent effect in the long term.[6–8] Other research found that some programs had a positive influence on adolescent tobacco-use.[9–12] A 2013 Cochrane review examined 49 randomized trials of school-based smoking prevention programs and found a significant effect in preventing uptake among students who had never smoked. The estimated difference in smoking uptake between intervention and control was 12%.[13,14] However, not all programs have been equally effective. Programs focusing on social competence, for example, were more effective than those providing information or focusing only on social influence.[13,14]. Success appears to depend on program content, intervention intensity, and program implementation.[12–15]

In applying for TUPE funding, grantees are encouraged to select from among empirically validated tobacco prevention curricula and to implement programs with fidelity.[5] Approved curricula include the following: Botvin’s Life Skills Training, Keepin’ It Real, Minnesota Smoking Prevention Program, Project Alert, Project Northland, Project Towards No Drug Use, Project Towards No Tobacco Use, Project Success, and Project SPORT. The TUPE recipients are required to implement these programs in accordance with fidelity guidelines and are required to submit progress reports to demonstrate program implementation.

Consistent with good practice and legislative statute, the TUPE program is subject to periodic evaluation to determine whether program goals are being met.[16] A past evaluation found that TUPE-funded schools were more likely to provide school-wide tobacco prevention education and that their students were more likely to report exposure to anti-tobacco education programs.[3] However, it also found a positive association between TUPE funding and tobacco use; rates were higher in TUPE-funded schools, a fact that might reflect pre-existing differences in the characteristics of schools that received funding.[3] Another study examined changes in tobacco use over time to determine impact of TUPE activities. It found that greater program implementation was associated with a non-significant decrease in tobacco use over time.[4]

Previous TUPE evaluations have not addressed emerging tobacco products, such as e-cigarettes. Yet, as use of conventional tobacco (e.g., cigarettes) declines and use of alternate forms of tobacco increases among adolescents,[17] it is important to address how these products are incorporated into prevention efforts. Therefore, this research explores the following questions for both conventional and alternative tobacco products: (1) are TUPE-funded schools doing more tobacco education than non-TUPE-funded schools? and (2) are TUPE-funded schools having better outcomes than non-TUPE-funded schools?

## Methods

### Data sources

This study utilized two data sources: (1) 2016 California Educator Tobacco Survey (CETS), and (2) the 2015–2016 California Student Tobacco Survey (CSTS). The sampling plan and measures for each survey are discussed below. Participants for the CETS provided informed consent electronically; for the CSTS parental consent requirements for minors varied according to school district policy. Most districts allowed opt-out consent from parents, while others required parents to opt-in. Students read the assent and confirmed electronically. Projects were approved by the University of California, San Diego’s Human Research Protections Program (150978, 170787) and the Committee for the Protection of Human Subjects (CPHS) of the California Health and Human Services Agency (CHHSA), (15-07-2105, 15-04-1992).

### California Educator Tobacco Survey (CETS), 2016

This survey was fielded April-August 2016 and collected data from California educators on tobacco education and prevention efforts at their schools. School selection criteria was identical to that used for CSTS (described below). Of 2,372 eligible schools, 1,000 were randomly selected for the sample. E-mail addresses were available for 867 schools; 176 schools had software blocking e-mail delivery, leaving a final sample of 691 schools (241 Tier 2, 78 Tier 1, and 372 non-TUPE). Eligible survey participants included administrators (principals, assistant principals, vice principals), teachers, and additional staff that interact with students. Administrative office staff, school psychologists, special education teachers, or staff having minimal interaction with students were excluded. Potential respondents were contacted via email and incentivized to complete the online survey with a chance to win one of several $100 gift cards (odds of winning = 1/120). In total, e-mails were sent to 50,700 unique addresses and educators were sent up to 7 reminders. The final sample included 3,564 individuals (7.0% of those invited to participate) representing 85.8% of the 691 schools with an average of 6.0 respondents per school (ranging from 1–28). Multiple responses from the same school were averaged to get an overall response from each school.

### California Student Tobacco Survey (CSTS), 2015–2016

The 2015–2016 CSTS collected data from California middle and high school students (grades 8, 10, and 12) on exposure to, attitudes about, and use of tobacco products. The CSTS uses a two-stage cluster sampling design where the school is the primary sampling unit and the classroom is the secondary sampling unit. Sampling was based on enrollment data provided by the California Department of Education (CDE). Schools were ineligible if they had fewer than 49 students per grade, were non-public or classified as special education, alternative, continuation, etc., or if students attended fewer than three days/week. From among those eligible (1,315 middle and 1,057 high schools), schools were selected proportionate to the number of students in the 12 regions used in the adult California Tobacco Survey.[18] Schools in less densely populated regions, those with a higher proportion of African American students, and those with Tier 2 TUPE funding were oversampled; the sample included 53 middle schools and 211 high schools. Response rates were 51% for middle schools (27 of 53) and 43% for high schools (90 of 211); the overall response rate was 44.3%. Schools were encouraged to survey all students in the grade. For those unable to do so, a second stage of sampling included randomly selecting 3–5 classes per grade for participation. The total number of students in the sample was 47,981.

### Measures

Provision of anti-tobacco educational programs was assessed on the CETS with questions about whether the school participated in specific health education programs (Red Ribbon Week, health fairs, Kick Butts day, etc.), whether tobacco prevention efforts were separate or part of an overall anti-drug program, and how well the school prepared staff to address tobacco use (“not well at all” vs “fairly well” or “extremely well”). Educators rated statements about perceived effectiveness of the tobacco-education programs, their confidence in talking to students about tobacco use, staff knowledge of how to prevent use, and enforcement of school tobacco policies. In the analyses “strongly” or “somewhat” agree were coded as “agree,” and “somewhat” or “strongly” disagree were coded as “disagree”.

To assess program outcomes, we examined CSTS data on student exposure to school-based tobacco education, use of traditional and emerging tobacco products, and perceptions about the health consequences of passive exposure to cigarette smoke and e-cigarette vapor. Current use was defined as use in the past 30 days. This study defines tobacco use in two ways: (1) use of cigarettes, little cigars or cigarillos (LCC), kreteks, cigars, hookah, or smokeless tobacco and (2) use of any of these products plus e-cigarettes. These definitions reflect the ongoing controversy in the field regarding whether e-cigarettes, despite FDA’s recent ruling, should be labelled as a tobacco product or regarded as a separate product.[17,19–23]

TUPE-funded schools were defined as schools that received money from the state to fully implement the TUPE program (i.e. Tier 2). Schools in districts that received Tier 1 funding were grouped with schools that received no funding as non-TUPE-funded schools since Tier 1 schools (n = 66) receive minimal funding and none for targeted educational programs.

### Analytic strategy

Bivariate analyses were conducted using chi-square to compare TUPE-funded and non-TUPE-funded schools on demographic characteristics as well as tobacco-related educational programs, tobacco-use policies, and norms from both the educator and student perspective. Comparisons of TUPE and non-TUPE schools were made at the school level for CETS (Tables 1–3) and the student level for CSTS (Tables 4–7). Hierarchical logistic regression modeling using the CSTS data (Table 7) was conducted with SAS GLIMMIX where students (level-1) were nested within schools (level-2). Of specific focus was the association between TUPE funding (level-2) and current tobacco use by middle and high school students (level-1), after controlling for student-level covariates (gender, age, grade, race/ethnicity, amount of pocket money, grades received in school, and school days missed) and size of school, a school-level covariate. Odds ratios and 95% confidence intervals were calculated for the models with two different outcome measures: the first predicted current tobacco use excluding e-cigarettes and the second predicted current tobacco use including e-cigarettes. CSTS results were weighted by 2015–16 school enrollment data obtained from CDE. Statistical analyses were conducted using SAS, version 9.4.

**Table 1:** Description of teachers/staff and knowledge of TUPE funding status, school-level.

	TUPE N = 208^a^ % (95% CI)	Non-TUPE N = 385^b^ % (95% CI)
**Which of the following BEST describes your position?**		
Teacher	78.4 (74.6–82.2)	81.2 (78.5–83.9)
Counselor	9.4 (6.8–11.9)	6.3 (4.8–7.9)
School Administrator	5.9 (3.8–8.0)	5.3 (3.7–6.9)
TUPE Advisor, Coordinator, or Specialist	1.3 (0.5–2.1)	0.8 (0.1–1.4)
After-school staff	0.1 (0.0–0.3)	0.1 (0.0–0.3)
Paraprofessional	1.4 (0.0–2.8)	1.1 (0.5–1.8)
Other	3.5 (1.5–5.6)	5.2 (3.6–6.7)
**Which grades, if any, do you work with?**		
6	17.9 (13.4–22.5)	14.2 (11.1–17.2)
7	32.3 (26.6–38.0)	26.8 (22.7–30.8)
8	31.6 (25.9–37.2)	27.3 (23.3–31.3)
9	49.9 (44.3–55.5)	53.4 (49.4–57.4)
10	53.3 (47.4–59.2)	56.8 (52.6–61.0)
11	55.6 (49.6–61.6)	60.6 (56.4–64.8)
12	56.3 (50.2–62.3)	60.2 (56.0–64.5)
**What subject(s) do you teach, if any?**		
English	28.1 (24.2–32)	26.9 (24–29.9)
Math	19.8 (16.3–23.3)	20.3 (17.6–23.0)
Science	26.8 (22.8–30.8)	27.2 (24.2–30.2)
History and Social Sciences	18.2 (14.9–21.6)	20.7 (17.8–23.5)
World Languages	6.2 (4.1–8.3)	6.4 (4.8–7.9)
Physical Education	5.3 (3.4–7.3)	5.8 (4.3–7.3)
Technology	7.2 (4.9–9.6)	5.4 (3.9–6.9)
Electives	27.0 (23.4–30.6)	27.2 (24.2–30.2)
**How long have you worked in education?**		
1–5 years	13.2 (10.5–16.0)	13.8 (11.6–16.0)
6–10 years	18.1 (14.6–21.5)	19.6 (16.9–22.4)
11–15 years	20.3 (16.9–23.7)	19.7 (17.2–22.1)
More than 15 years	48.4 (43.9–52.9)	46.9 (43.7–50.1)
**What kind of funding does your school currently receive from California’s Tobacco Use Prevention Education (TUPE) program?**		
I do not know	82.6 (79.4–85.9)	84.7 (82.4–87.0)

Source: California Educator Tobacco Survey, 2016.

Notes:

^a^1,185 respondents.

^b^2,379 respondents.

**Table 2:** Programs and positive development in non-TUPE vs. TUPE schools, school-level.

	TUPE N = 208^a^ %(95% CI)	Non-TUPE N = 385^b^ %(95% CI)
**Does your school participate in or offer any of the following:**	% Yes	% Yes
Red Ribbon Week	68.1 (63.1–73.1)	65.4 (61.5–69.3)
Health Fairs	38.5 (34.0–42.9)	36.6 (33.2–40.0)
Groups to help students quit smoking	22.3 (18.2–26.5)	9.4 (7.6–11.3)
Out-of-school clubs to prevent tobacco use	24.1 (20.0–28.2)	11.4 (9.2–13.6)
Great American Smokeout	22.3 (18.1–26.4)	11.7 (9.5–13.9)
Kick Butts Day	17.6 (13.7–21.4)	3.9 (2.5–5.4)
**How much of a priority does your school place on the following tobacco prevention efforts?**	% 4–5 Very High Priority	% 4–5 Very High Priority
Target at-risk youth	29.8 (25.7–33.9)	19.3 (16.8–21.8)
Peer-to-peer programs	18.9 (15.1–22.6)	10.3 (8.3–12.3)
School wide activities	20.0 (16.4–23.7)	10.0 (8.1–12.0)
Referring tobacco users to cessation services such as the California Smokers’ Helpline	13.6 (10.5–16.6)	6.3 (4.8–7.7)
**At your school, how are tobacco prevention efforts presented?**		
As part of an overall drug prevention program	41.2 (37–45.4)	41.6 (38.4–44.8)
Separately	23.3 (19.6–27.0)	13.6 (11.3–15.8)
I do not know	35.5 (31.2–39.8)	44.8 (41.6–48.0)
**How seriously does your school take its tobacco-prevention efforts?**		
Somewhat/Not at all seriously	55.9 (51.3–60.5)	66.4 (63.2–69.6)
Seriously/Extremely seriously	44.1 (39.5–48.7)	33.6 (30.4–36.8)
**How well has your school prepared you to address tobacco use among students?**		
Not well at all	53.4 (48.9–57.9)	68.4 (65.4–71.5)
Fairly/Extremely well	46.6 (42.1–51.1)	31.6 (28.5–34.6)

Source: California Educator Tobacco Survey, 2016.

Notes:

^a^1,185 respondents.

^b^2,379 respondents.

**Table 3:** Tobacco control perceptions in non-TUPE vs. TUPE schools.

How much do you agree or disagree with the following statements about…	TUPE N = 208^a^ %(95% CI)	Non-TUPE N = 385^b^ %(95% CI)
	% Agree (strongly or somewhat)	% Agree (strongly or somewhat)
**Tobacco (cigarettes, little cigars, or cigarillos)**		
A. I always enforce my school’s rules against using **tobacco** on school grounds.	97.2 (95.7–98.7)	98.0 (97.4–98.6)
B. I would feel confident talking to students about the negative aspects of **tobacco** use.	92.3 (90.0–94.5)	91.9 (90.4–93.5)
C. Faculty and staff at this school know how to help students avoid **tobacco**.	62.7 (58.4–67.0)	51.9 (48.6–55.1)
D. Tobacco prevention efforts at my school are effective at helping students avoid **tobacco**.	53.0 (48.5–57.4)	38.4 (35.1–41.7)
**E-cigarettes**		
A. I always enforce my school’s rules against using **e-cigarettes** on school grounds.	96.2 (94.7–97.7)	95.8 (94.6–96.9)
B. I would feel confident talking to students about the negative aspects of **e-cigarettes** use.	79.8 (76.5–83.2)	78.3 (75.8–80.8)
C. Faculty and staff at this school know how to help students avoid **e-cigarettes**.	44.1 (39.7–48.4)	35.4 (32.2–38.6)
D. Tobacco prevention efforts at my school are effective at helping students avoid **e-cigarettes**.	45.2 (40.5–50.0)	30.0 (26.9–33.1)

Source: California Educator Tobacco Survey, 2016.

Notes:

^a^1,185 respondents.

^b^2,379 respondents.

**Table 4:** Sample characteristics (CSTS).

	TUPE (N = 27892)		Non-TUPE (N = 20089)	
	%	95% CI	%	95% CI
**Gender**				
Male	49.1	(48.1–50.0)	49.6	(48.6–50.6)
Female	50.9	(50.0–51.9)	50.4	(49.4–51.4)
**Age**				
11–13	19.8	(18.9–20.7)	22.0	(21.0–22.9)
14–16	46.5	(45.5–47.4)	43.4	(42.4–44.4)
17–19	33.7	(32.9–34.6)	34.6	(33.7–35.5)
**Grade**				
8	28.5	(27.5–29.5)	29.7	(28.7–30.7)
10	38.0	(37.1–38.8)	36.0	(35.0–37.0)
12	33.6	(32.7–34.4)	34.3	(33.4–35.2)
**Race**				
NH-White	16.2	(15.5–16.8)	16.1	(15.4–16.8)
NH-Black	3.9	(3.5–4.3)	3.3	(2.9–3.7)
Hispanic	49.7	(48.7–50.6)	61.5	(60.5–62.5)
NH-Asian	16.2	(15.5–16.9)	7.9	(7.3–8.4)
NH-AI/AN	0.3	(0.2–0.4)	0.3	(0.2–0.4)
NH-NHOPI	1.1	(0.9–1.2)	0.7	(0.5–0.8)
NH-Other	2.9	(2.6–3.2)	2.2	(1.9–2.5)
NH-Multiple race	9.8	(9.3–10.4)	8.0	(7.4–8.6)
**Pocket money**				
$0	40.0	(39.1–40.9)	36.0	(35.0–37.0)
$1–10	18.8	(18.1–19.6)	21.3	(20.5–22.2)
$11–50	26.7	(25.8–27.5)	27.3	(26.4–28.2)
$50+	14.5	(13.9–15.1)	15.3	(14.7–16.0)
**Academic Performance**				
A's & B's	56.4	(55.5–57.3)	51.9	(50.9–53.0)
B's & C's	29.1	(28.2–30.0)	32.2	(31.2–33.1)
C's & D's	10.0	(9.4–10.5)	10.9	(10.3–11.6)
D's & F's	3.6	(3.3–4.0)	3.9	(3.5–4.3)
No letter grade	0.9	(0.7–1.1)	1.1	(0.9–1.3)
**Days missed school**				
0	43.8	(42.8–44.7)	40.8	(39.7–41.8)
1–5	45.6	(44.7–46.6)	48.4	(47.4–49.5)
6+	10.6	(10.0–11.2)	10.8	(10.2–11.4)

Source: California Student Tobacco Survey (CSTS), 2015–2016

**Table 5:** Student exposure to advertising in the community (CSTS).

	TUPE % (95% CI)	Non-TUPE % (95% CI)
**Last 30-day exposure to advertising FOR cigarettes**	N = 27553	N = 19892
Social media	30.2 (29.4–31.1)	32.1 (31.1–33.1)
TV	39.5 (38.6–40.5)	42.0 (41.0–43.0)
Magazines	19.3 (18.5–20.0)	17.5 (16.7–18.3)
Gas stations or convenience stores	38.6 (37.7–39.5)	37.2 (36.2–38.2)
Vape or tobacco shops	28.8 (27.9–29.6)	28.2 (27.2–29.1)
Seen any	68.3 (67.4–69.2)	69.7 (68.7–70.6)
**Last 30-day exposure to advertising AGAINST cigarettes**	N = 27612	N = 19930
Social media	55.2 (54.2–56.1)	55.8 (54.8–56.8)
TV	67.3 (66.4–68.2)	70.4 (69.4–71.3)
Magazines	17.7 (16.9–18.4)	17.8 (17.0–18.6)
Gas stations or convenience stores	15.5 (14.9–16.2)	17.5 (16.7–18.3)
Vape or tobacco shops	6.6 (6.1–7.0)	7.1 (6.6–7.7)
Seen any	81.8 (81.1–82.5)	82.7 (81.9–83.5)
**Last 30-day exposure to advertising FOR e-cigarettes**	N = 27501	N = 19856
Social media	23.7 (23.0–24.5)	24.3 (23.4–25.2)
TV	28.8 (27.9–29.7)	30.5 (29.5–31.4)
Magazines	13.3 (12.7–13.9)	12.9 (12.2–13.6)
Gas stations or convenience stores	22.4 (21.6–23.1)	21.6 (20.8–22.5)
Vape or tobacco shops	22.7 (21.9–23.4)	21.7 (20.8–22.5)
Seen any	52.8 (51.9–53.8)	53.3 (52.3–54.3)
**Last 30-day exposure to advertising AGAINST e-cigarettes**	N = 27492	N = 19841
Social media	24.3 (23.5–25.1)	23.9 (23.0–24.8)
TV	33.9 (33.0–34.8)	34.6 (33.6–35.6)
Magazines	9.2 (8.6–9.7)	9.0 (8.4–9.6)
Gas stations or convenience stores	6.3 (5.8–6.7)	6.8 (6.3–7.4)
Vape or tobacco shops	4.1 (3.8–4.5)	4.6 (4.2–5.0)
Seen any	44.4 (43.5–45.4)	45.1 (44.0–46.1)
Last 30-day exposure to advertising FOR LCC*	N = 15133	N = 11636
Social media	15.4 (14.6–16.3)	17.2 (16.2–18.2)
TV	20.4 (19.4–21.3)	23.1 (21.9–24.2)
Magazines	9.2 (8.5–9.9)	8.9 (8.2–9.7)
Gas stations or convenience stores	15.8 (14.9–16.6)	16.0 (15.0–16.9)
Vape or tobacco shops	12.7 (11.9–13.5)	12.3 (11.5–13.1)
Seen any	36.2 (35.1–37.4)	39.0 (37.8–40.3)
Last 30-day exposure to advertising AGAINST LCC*	N = 15127	N = 11631
Social media	20.8 (19.9–21.8)	22.0 (20.9–23.1)
TV	28.6 (27.6–29.7)	30.6 (29.4–31.8)
Magazines	9.0 (8.3–9.6)	9.1 (8.4–9.9)
Gas stations or convenience stores	7.7 (7.1–8.4)	8.7 (8.0–9.5)
Vape or tobacco shops	4.3 (3.8–4.8)	4.9 (4.3–5.5)
Seen any	36.8 (35.7–38.0)	39.7 (38.5–41.0)

Source: California Student Tobacco Survey (CSTS), 2015–2016.

Notes:

*little cigars and cigarillos.

**Table 6:** Classes in TUPE vs. Non-TUPE schools (CSTS).

	TUPE % (95% CI)	Non-TUPE % (95% CI)
**Education on Active use**		
In the last 12 months, did any of your classes or school activities talk about the harmful effects of using the following products?	N = 27569	N = 19923
Cigarettes	49.9 (49.0–50.8)	38.8 (37.8–39.8)
Cigars (cigars, little cigars, cigarillos)	30.5 (29.6–31.4)	21.9 (21.0–22.7)
Hookah	25.4 (24.5–26.3)	16.9 (16.2–17.7)
E-cigarettes	32.7 (31.8–33.6)	20.6 (19.8–21.4)
Smokeless tobacco (chew, dip, snuff or snus)	36.2 (35.2–37.1)	25.5 (24.6–26.4)
**Education on Passive exposure**		
In the last 12 months, did any of your classes or school activities talk about the harmful effects of the following?	N = 27543	N = 19937
Smoke from cigarettes	45.8 (44.9–46.8)	37.0 (36.0–38.0)
Vapor from e-cigarettes	23.1 (22.2–23.9)	15.4 (14.7–16.2)

Source: California Student Tobacco Survey (CSTS), 2015–2016

**Table 7:** Modeling outcomes: Current tobacco use, controlling for covariates.

	Current Tobacco Use (Excluding e-cigarette)		Current Tobacco (Including e-cigarette)	
	OR	95% CI	OR	95% CI
**School Level (N = 117)**				
TUPE Status				
Non-TUPE	ref.	1	ref.	1
TUPE	0.79	(0.63–1.00)	0.82	(0.70–0.96)
School Size				
<1000 students	ref.	1	ref.	1
1000–1999 students	0.72	(0.53–0.98)	0.72	(0.59–0.89)
2000+ students	0.77	(0.56–1.07)	0.80	(0.64–1.00)
**Student Level (N = 46789)**				
Gender				
Male	ref.	1	ref.	1
Female	0.77	(0.75–0.78)	0.81	(0.80–0.82)
Age				
unit change from school mean	1.28	(1.27–1.30)	1.22	(1.21–1.23)
Grade				
8	ref.	1	ref.	1
10	3.68	(3.08–4.40)	3.39	(3.01–3.82)
12	4.07	(3.40–4.88)	3.83	(3.39–4.33)
Race/Ethnicity				
NH-White	ref.	1	ref.	1
NH-Black	0.74	(0.70–0.78)	0.65	(0.62–0.68)
Hispanic	0.90	(0.88–0.92)	0.88	(0.86–0.90)
NH-Asian	0.38	(0.37–0.40)	0.46	(0.45–0.48)
NH-AI/AN	1.92	(1.72–2.15)	1.57	(1.41–1.74)
NH-NHOPI	0.89	(0.82–0.97)	0.83	(0.77–0.89)
NH-Other	1.46	(1.39–1.53)	1.16	(1.11–1.21)
NH-Multiple race	1.01	(0.98–1.05)	1.00	(0.97–1.02)
Pocket money				
$0	ref.	1	ref.	1
$1–10	1.26	(1.23–1.29)	1.25	(1.23–1.28)
$11–50	1.79	(1.75–1.82)	1.86	(1.83–1.90)
$50+	3.50	(3.43–3.58)	3.30	(3.24–3.37)
Academic Performance				
A's & B's	ref.	1	ref.	1
B's & C's	1.45	(1.43–1.48)	1.43	(1.41–1.45)
C's & D's	2.12	(2.07–2.17)	1.86	(1.83–1.90)
D's & F's	3.38	(3.28–3.49)	2.80	(2.72–2.88)
No letter grade	1.85	(1.74–1.96)	1.50	(1.42–1.59)
Days missed school				
0	ref.	1	ref.	1
1–5	1.26	(1.24–1.28)	1.27	(1.25–1.29)
6+	2.58	(2.52–2.63)	2.39	(2.34–2.44)

*Source*: California Student Tobacco Survey (CSTS), 2015–2016.

NH = Non-Hispanic.

AI/AN = American Indian/Alaskan Native.

NHOPI = Native Hawaiian and Other Pacific Islanders.

## Results

### California Educator Tobacco Survey, 2016

Table 1 presents respondent characteristics for the educator survey. The majority of respondents were teachers, with broad representation of subjects taught and greater representation of high-schools than middle schools. Nearly half of respondents had worked in education over 15 years. There were no significant differences between TUPE and non-TUPE school respondents in terms of staff position, grades taught, subjects taught, or time in education. Respondents from TUPE and non-TUPE schools were equally unaware of their school’s TUPE funding status (82.6% in TUPE-funded schools compared to 84.7% in non-TUPE-funded schools, p = 0.30).

Respondents were asked to identify types of health promotion and tobacco prevention activities conducted at their school (Table 2). There were no differences based on TUPE funding on non-tobacco-specific health promotion activities such as Red Ribbon Week (p = 0.41) and health fairs (p = 0.51). However, TUPE schools reported higher rates of tobacco-specific activities, such as groups to help students quit smoking (22.3% vs. 9.4%, p < .0001), out-of-school clubs to prevent tobacco use (24.1% vs. 11.4%, p < .0001), and participating in the Great American Smokeout (22.3% vs. 11.7%, p < .0001) or Kick Butts Day (17.6% vs. 3.9%, p < .0001). TUPE school respondents more often reported that their school placed high or very high priority on specific tobacco-prevention efforts such as targeting at-risk youth (29.8% vs. 19.3%, p < .0001), providing peer-to-peer programs (18.9% vs. 10.3%, p < .0001), holding schoolwide activities (20.0% vs. 10.0%, p < .0001), and referring tobacco users to cessation services such as the California Smokers’ Helpline (13.6% vs. 6.3%, p < .0001). In addition, TUPE school respondents were more likely to report that tobacco prevention efforts were presented separately from an overall drug program (23.3% vs. 13.6%, p < .0001), that their school takes tobacco-prevention efforts seriously or extremely seriously (44.1% vs. 33.6%, p < .0001), and that they were extremely well or fairly well prepared to address tobacco use among students (46.6% vs. 31.6%, p < .0001).

Table 3 presents respondent perceptions of the tobacco control efforts at their school for traditional tobacco products and e-cigarettes. Nearly all respondents (>95%) reported that they always enforce school rules against using tobacco and e-cigarettes and more expressed confidence talking to students about the negative aspects of tobacco than of e-cigarettes (92.0% vs 78.8%, p<0.001), regardless of funding status. When asked more generally about “the faculty and staff at this school” or “the tobacco prevention efforts at my school,” there were significant differences based on funding status. Respondents at TUPE-funded schools were more likely to report that faculty and staff at their school know how to help students avoid tobacco (62.7% vs. 51.9%, p < .0001) and e-cigarettes (44.1% vs. 35.4%, p = .002). They were also more likely to report that their schools’ tobacco prevention efforts (53.0% vs. 38.4%, p < .0001) and e-cigarette prevention efforts (45.2% vs. 30.0%, p < .0001) are effective.

### California Student Tobacco Survey (CSTS), 2015–2016

**Fig 1:**
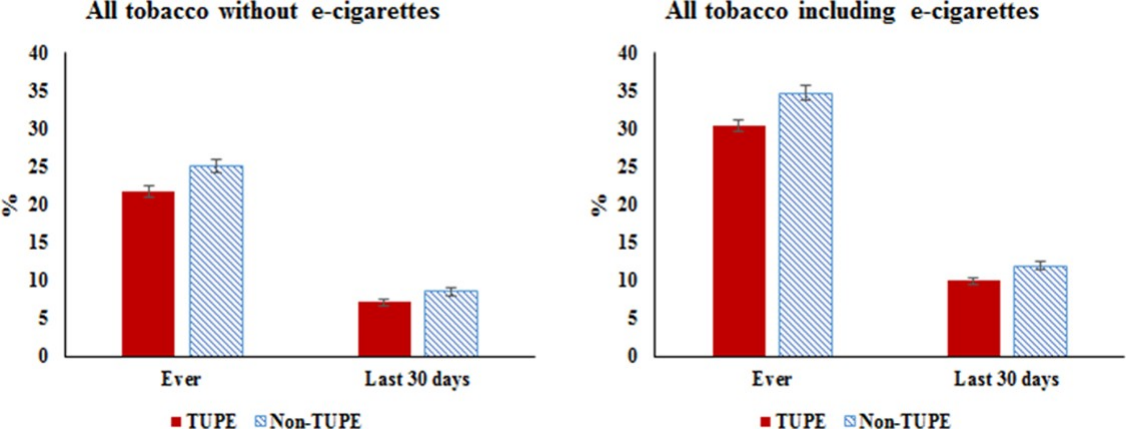
Prevalence of tobacco use in TUPE vs. non-TUPE schools. Source: California Student Tobacco Survey (CSTS), 2015–2016.

Tables 4–7 and Fig 1 present analyses of student data from the CSTS. Table 4 compares the sample characteristics of CSTS respondents finding no significant differences by TUPE status on gender or grade. However, students from TUPE schools were less likely to be Hispanic (49.7% vs. 61.5%, p < .0001) and more likely to be non-Hispanic Asian (16.2% vs. 7.9%, p < .0001) than those from non-TUPE schools. Other significant differences included TUPE school respondents being more likely to report being age 14–16 (46.5% vs. 43.4%) having no pocket money (40.0% vs. 36.0%, p < .0001), receiving A’s and B’s (56.4% vs. 51.9%, p < .0001), and missing zero days of school (43.8% vs. 40.8%, p < .0001).

Table 5 presents exposure to anti-tobacco messages outside of the school environment. Overall, students in TUPE-funded and non-TUPE-funded schools reported similar levels of exposure to advertising against cigarettes (p = 0.32), e-cigarettes (p = 0.38), and LCCs (p < .001). There were some differences in the channel of exposure by funding; students at TUPE-funded schools were less likely to report seeing advertising against cigarettes on television (p = .0001), at gas stations or convenience stores (p = .002), or at vape or tobacco shops (p = 0.024). Likewise, reports of exposure to advertisements that promoted cigarettes, e-cigarettes, or LCC were similar across funding with some differences across channel of exposure. Students at TUPE-funded schools were less likely to report advertising of cigarettes (p = .002), e-cigarettes (p = 0.021 and LCCs (p = 0.0004) on television, but more likely to report exposure to cigarette advertising in magazines (p = 0.018) than students at non-TUPE-funded schools.

As with the educator survey, students at TUPE schools reported higher levels of school activities related to the harmful effects of tobacco and e-cigarette use. Table 6 shows that students at TUPE schools were more likely to report that their classes or school discussed harmful effects of cigarettes (49.9% vs. 38.8%), cigars (30.5% vs. 21.9%), hookah (25.4% vs. 16.9%), e-cigarettes (32.7% vs. 20.6%), smokeless tobacco (36.2% vs. 25.5%), smoke from cigarettes (45.8% vs. 37.0%), and vapor from e-cigarettes (23.1% vs. 15.4%); all comparisons with non-TUPE schools were significant at p < .0001.

Fig 1 presents prevalence of tobacco use in TUPE vs. non-TUPE-funded schools using both the definition that includes and that excludes e-cigarettes. In either case, TUPE-funded schools had lower rates of tobacco use. Rates of ever using tobacco (including e-cigarettes) were 30.4% and 34.6% for TUPE-funded and non-TUPE-funded schools, respectively (p < .0001). Likewise, rates of current use of tobacco (including e-cigarettes) was lower for TUPE-funded schools than for non-TUPE-funded schools (9.9% vs. 12.5%, p < .0001). These patterns persisted when e-cigarettes were excluded from the definition of tobacco. Ever use of tobacco (excluding e-cigarettes) was 21.7% [95% CI 20.9–22.4] and current use was 7.1% [95% CI 6.6–7.5] at TUPE-funded schools compared to 25.2% [95% CI 24.3–26.0] ever use and 8.6% [95% CI 8.0–9.1] current use at non-TUPE-funded school.

Table 7 presents the multilevel logistic regression analyses modeling current tobacco use (excluding and including e-cigarettes). Students in TUPE-funded schools had a lower adjusted odds of current tobacco use than students in non-TUPE schools (OR = 0.79, 95% CI = 0.63–1.00 excluding e-cigarettes and OR = 0.82, 95% CI = 0.70–0.96 including e-cigarettes). In addition to TUPE status, other characteristics associated with lower odds of tobacco-use included larger school size, being female, having less pocket money, getting higher grades, and missing fewer days of school. There were also differences in adjusted odds of using tobacco by race/ethnicity where non-Hispanic blacks, Hispanics, and non-Hispanic Asians had lower rates of tobacco use (both excluding or including e-cigarette use) than non-Hispanic whites, while non-Hispanic American Indians/Alaska natives had higher rates of use.

## Discussion

### Summary

This study found that TUPE-funded schools were more likely to provide tobacco-specific health education programs, prioritize tobacco-prevention, and prepare educators to address tobacco-use. There is converging evidence from the two surveys. There was no difference in educators’ report of general health education programs based on TUPE funding but greater report of tobacco-specific education activities in TUPE-funded schools. The same pattern was found in the student survey where there was no difference by funding on reports of being exposed to anti-tobacco campaigns outside of the school context, yet students at TUPE-funded schools were more likely to report receiving anti-tobacco messages at school. Moreover, students at TUPE-funded schools were less likely to use tobacco products than those at non-funded schools, even controlling for demographic differences. Taken together, these results show that the TUPE program was associated with an increase in students’ awareness of the dangers of tobacco and a decrease in the use of tobacco. These results also suggested that both TUPE and non-TUPE-funded schools were doing a better job addressing cigarette use than addressing other emerging tobacco products such as e-cigarettes. While our study design precludes any causal inferences, our findings would support the conclusion that the TUPE program is effective in influencing students’ awareness and smoking behavior.

### Limitations

There are limitations to such an interpretation of these results. First, schools were not randomly assigned to TUPE funding, therefore other factors might explain the association between funding and tobacco use. Schools that received funding might have been those that had a greater perceived need for these programs, or conversely, those without funding might be those that had fewer resources to put toward grant writing. Even so, the multilevel logistic regression models show that TUPE funding is associated with lower tobacco use, even controlling for demographic and school characteristics. Secondly, the cross-sectional nature of the data and the lack of baseline data on tobacco consumption limits any direct inference as to the impact of the TUPE program. In addition, this analysis was not able to control for the length of time a school had participated in the TUPE program. It is possible that schools with more recent funding may differ from schools with a longer history in the TUPE program in ways that are not captured in this analysis. A final limitation is the potential for non-response bias in the CETS due to the low response rate at the level of individual educators (7.0%). However, the response rates for TUPE and non-TUPE schools were similar, suggesting similarity in reasons for nonresponse for educators from both TUPE and non-TUPE schools. At the same time, the school level response rate was high (85.4%) and more importantly, information from educators agrees with student reports.

### Implications of the findings

This research addressed two questions: (1) are TUPE-funded schools doing more tobacco education than non-TUPE-funded schools, and (2) are TUPE-funded schools having better outcomes than non-TUPE-funded schools? A discussion of each is presented below.

Our results demonstrated that TUPE-funded schools provide more education about tobacco than non-TUPE schools. At TUPE-funded schools educators feel that tobacco prevention is given higher priority, that their school’s tobacco prevention programs are effective, and feel better prepared to address tobacco use among students. Even so, over two thirds of educators in non-TUPE-funded schools and over half in funded schools did not feel the school had prepared them sufficiently to address tobacco use with students. And less than one-third of respondents at funded school reported that tobacco use was a high priority. These results indicate that there is considerable room to improve the TUPE program. Given the many competing priorities that schools face, schools may need greater incentives to prioritize tobacco prevention efforts.

It is clear from this study that educators feel less comfortable addressing the health consequences of emerging products than of standard tobacco, regardless of funding. New vaping products (e.g., JUULs and Suorin) look very different from earlier models of vapes making them more likely to be used by youth during class without a teacher even knowing.[24] As these products continue to evolve and proliferate, it is increasingly important to educate school staff about them. As a result, CDE might want to strengthen the training and support for educators related to these products.

Student results confirm that TUPE-funded schools are providing more education to adolescents on the harmful effects of tobacco products than are non-TUPE-funded schools. The literature suggests that school-based tobacco prevention programs can be effective in preventing the initiation of tobacco products if the programs are sufficiently intense and well implemented.[4,11–15] Therefore, if the program is well implemented, we would expect students would be less likely to use tobacco products. Our research shows that students at TUPE-funded schools are indeed less likely to use tobacco products than students at non-funded schools. Since the data are cross-sectional, it is not possible to definitively state that the lower rate of tobacco use is the direct result of the TUPE-program. However, it is consistent with research by Park et al. who found a trend (non-significant) between TUPE funding and reduced tobacco-use among students using a longitudinal design.[4] A randomized trial should be considered for future research to establish causality between TUPE-funding and subsequent tobacco use.[4]

The educator survey revealed a number of similarities between TUPE and non-TUPE-funded schools that strengthen our confidence in the differences we report. First, educators were very unlikely to know their school’s funding status. This makes it unlikely that differences found were due to biased reporting by staff at TUPE-funded schools. Second, educators were equally likely to report that their schools participated in general health promotion programs such as Red Ribbon Week and health fairs, regardless of funding. Likewise, they were equally likely to report enforcing school tobacco policies and having the confidence to address negative aspects of tobacco use with students. Yet, educators differed in their report of tobacco-specific education based on TUPE funding.

The student survey also found important similarities between TUPE and non-TUPE-funded schools that corroborate the conclusions. Students reported very similar exposure to counter-marketing against tobacco products outside of the school environment and across five different marketing outlets, regardless of TUPE funding. Yet, as with the educator survey, there were notable differences in reported exposure to classes and school activities that were specific to tobacco. Students from TUPE-funded schools reported being exposed at higher rates to classes or school activities on the harmful effects of all the tobacco products listed and on secondhand exposure to cigarettes and e-cigarettes. These reports from educators and students provide converging evidence that students in TUPE schools were exposed to more tobacco control activities at their school.[25]

## Conclusion

This study found TUPE-funded schools engaged in more tobacco prevention activities than non-TUPE-funded schools, which was associated with lower tobacco use among students at funded schools. This study also found that education and prevention efforts regarding emerging tobacco products need to be strengthened across all schools.
